# Persistence of onchocerciasis in villages in Enugu and Ogun states in Nigeria following many rounds of mass distribution of ivermectin

**DOI:** 10.1186/s12879-022-07811-7

**Published:** 2022-11-10

**Authors:** Uwem F. Ekpo, Obiora A. Eneanya, Edith N. Nwankwo, Islamiat Y. Soneye, Gary J. Weil, Peter U. Fischer, Obioma C. Nwaorgu

**Affiliations:** 1 Department of Pure and Applied Zoology, Federal University of Agriculture, Abeokuta, Ogun Nigeria; 2 Infectious Diseases Division, Washington University School of Medicine, St. Louis, MO USA; 3 Department of Parasitology and Entomology, Faculty of Biosciences, Nnamdi Azikiwe University, Awka, Anambra Nigeria; 4 Neglected Tropical Disease Control Unit, Ogun State Ministry of Health, Oke-Mosan, Abeokuta, Ogun Nigeria

**Keywords:** Onchocerciasis, Ivermectin, Mass drug administration, Nigeria

## Abstract

**Background:**

Onchocerciasis is endemic in most local government areas (LGAs) in Enugu and Ogun states. Most meso- and hyper-endemic LGAs have received many rounds of ivermectin mass drug administration (MDA). This study aimed to determine the current prevalence of onchocerciasis in villages in Enugu and Ogun states that were formerly highly endemic and to assess progress toward elimination of the infection in areas believed to be at high risk for persistence.

**Methods:**

Cross-sectional community surveys were conducted 8 to 12 months after the last round of MDA in 16 villages (6 in Enugu state and 10 in Ogun state) in individuals aged ≥ 18 years. Study participants were examined for the presence of palpable subcutaneous nodules. Skin snips from the posterior iliac crests were used to assess microfiladermia (Mf) prevalence and density.

**Results:**

643 subjects were palpated for nodules and 627 individuals (225 in Enugu state; 402 in Ogun state) provided skin snips. Nodule prevalence in the study villages ranged from 42 to 66.7% in Enugu state and from 0 to 25.0% in Ogun state. Mf prevalence in the Enugu and Ogun study villages ranged from 32 to 51.1% and 0 to 28.6%, respectively. Geometric mean skin Mf density in surveyed Enugu state villages ranged between 1 and 3.1 Mf/mg; these values were < 1 Mf/mg in all but one community in Ogun state villages.

**Conclusion:**

Results from this study show that onchocerciasis persists in adults in many villages in Enugu and Ogun states despite many prior rounds of ivermectin MDA. Prevalence was higher in villages surveyed in Enugu than in Ogun. Low Mf densities suggest the MDA program is working well to reduce disease, but more time will be required to reach the elimination goal.

**Supplementary Information:**

The online version contains supplementary material available at 10.1186/s12879-022-07811-7.

## Background

Onchocerciasis (also known as river blindness) is one of the neglected tropical diseases (NTDs) targeted by the World Health Organization (WHO) for elimination as a public health problem by 2030 [1]. The disease is caused by the filarial nematode *Onchocerca volvulus,* and it is transmitted by *Simulium* black flies. Female worms which live in subcutaneous nodules release microfilariae (Mf) that migrate through the skin and are picked up by the vector. Microfilariae are not only crucial for transmission; host inflammatory reactions to Mf cause severe morbidity including visual impairment, blindness, and severe dermatitis which can be disabling and stigmatizing [2]. Infected persons often experience negative social consequences and economic loss [3].

Rapid epidemiological mapping of onchocerciasis was carried out in Nigeria between 1994 and 1996 [4]. This mapping provided estimates of the populations living in high-risk areas and identified villages to be prioritized for ivermectin MDA. Subsequently, MDA commenced in 1997 utilizing local volunteers, known as community drug distributors (CDDs), for the distribution of ivermectin. This strategy has been an effective strategy for drug delivery [5]. Although ivermectin is effective for clearing Mf from the skin and for interrupting embryogenesis in the female worm, these effects are only temporary, and the treatment has little effect on adult worm viability [6, 7]. For this reason, repeated treatments are needed to suppress Mf to levels that do not support transmission for the reproductive life span of adult worms (> 10 years).

Nigeria has the largest at-risk population for onchocerciasis of any country in Africa. Historical mapping data showed that all 17 Local Government Areas (LGAs) in Enugu state and eight in Ogun state were endemic for onchocerciasis [8]. Some endemic foci in these states have received 14 to 26 rounds of ivermectin MDA for onchocerciasis, respectively. Also, since these states are co-endemic for lymphatic filariasis (LF) [9, 10], ivermectin plus albendazole MDA has been delivered annually in many LGAs since 2000. No data have been published in recent years on the current status of onchocerciasis or progress toward elimination in these states. The data reported here were collected during pilot surveys that were conducted to identify potential sites for clinical trials of new treatments for onchocerciasis, i.e., triple-drug therapy with ivermectin, diethylcarbamazine, and albendazole (IDA) [11] (see: https://dolfproject.wustl.edu/about/ida-for-onchocerciasis-studies/). They provide important information on the persistence of onchocerciasis in some areas of Nigeria that had high baseline endemicity despite the clear beneficial effects of extensive MDA with ivermectin.

## Methods

### Study areas

**Fig. 1:**
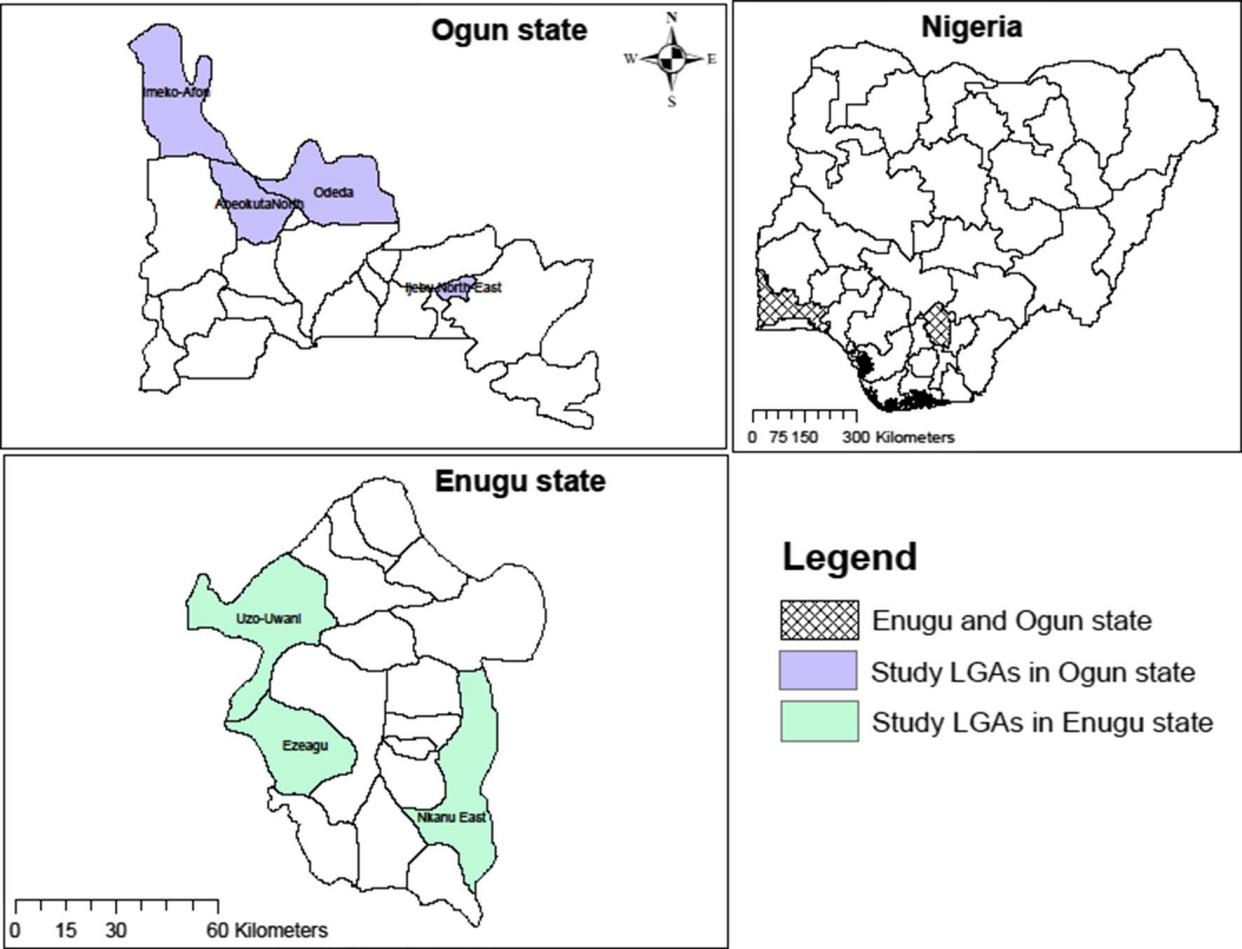
Map showing the location of study local government areas (LGAs) in Enugu and Ogun states, Nigeria

Enugu and Ogun states are located in the southeastern and southwestern geopolitical zones of Nigeria respectively (Fig. 1). These states have a combined population of about 7 million in 37 local government areas (LGAs, 17 in Enugu and 20 in Ogun). LGAs serve as implementation units (IU) for MDA programmes. The sites selected for this study lie within the rainforest belt of Nigeria, where villages are often located close to fast-flowing rivers that have suitable breeding sites for *Simulium* black flies [12]. The major rainy season is between April and July and the minor rainy season occurs between October to November. The mean annual temperatures in southern Nigeria range from 24 to 32 °C.

### Study design

The surveys were performed to identify potential sites for clinical trials of new treatments for onchocerciasis. The surveys also allowed the assessment of current nodule and Mf prevalence in villages following extensive MDA with ivermectin. Villages were selected based on guidance from health officials in Enugu and Ogun states to include areas that were previously classified as either meso- or hyperendemic for onchocerciasis.

#### Recruitment of study participants

Cross-sectional surveys were conducted in 16 villages (6 in Enugu and 10 in Ogun state) in selected LGAs between September 2020 and May 2021. A convenience sample of consenting individuals ≥ 18 years who were residents of the villages were eligible to participate. Before recruitment, the study objectives were discussed with participants and a signed informed consent to participate was obtained from all participants. An interpreter, for Igbo (in Enugu) and Yoruba (in Ogun) languages, was used if the participant did not speak or understand the English language. These surveys were conducted with help from community drug distributors (CDDs), local health officials and/or village heads, with supervision by the study coordinators. The surveys aimed to enrol 40 participants (both genders) per study site.

#### Nodule palpation

Individuals were palpated to check for the presence of nodules harbouring adult worms of *O. volvulus*. Subcutaneous nodules were firm, often flattened or bean-shaped, usually movable, non-tender palpable masses that were usually 0.5 to 10 cm centimetres in diameter [2]. They were distant from the usual locations of lymph nodes, (neck, axillae, and inguinal regions); palpation was performed over bony prominences (ribs, iliac crests, sacrum, and upper leg). Nodule presence was a binary finding (i.e., positive, or negative) for each participant. The number of nodules palpated per person was also noted and recorded. Demographic information for participants was also recorded.

#### Detection of *O. volvulus* microfilariae

Skin snips were obtained from participants with or without palpable nodules. The skin was cleaned with isopropyl alcohol wipes. Skin snips were taken from each posterior iliac crest using a sterile 2 mm Holth-type corneoscleral punch (Everhards GmbH, Meckenheim, Germany). Each skin snip was weighed with a digital balance. Snips were incubated in 100 μl phosphate-buffered saline for 24 h in flat-bottomed microtiter plates at room temperature. Emerging microfilariae were counted by bright-field microscopy (40x).

#### Data management and statistical analysis

Data were entered into Microsoft Excel 365 (Microsoft Corporation) spreadsheets and imported into IBM SPSS statistical software© (v 20.0, IBM Corporation, Armonk, NY) for analyses. Mf prevalence was expressed as a percentage (number of persons positive for Mf in either snip divided by number examined × 100). Mf intensity was expressed as the number of Mf per mg of skin snip. Chi-square was used to test the statistical significance of differences in Mf and nodule prevalence between study villages. A two-tailed *p*-value < 0.05 was considered statistically significant. The mean Mf/mg from the 2 skin snips were calculated per person to provide a single value of Mf intensity for each participant. Geometric mean Mf prevalence were calculated for each village. Geometric mean infection intensity was calculated with data from persons with Mf values > 0.

## Results

### Demographic and mass drug administration data of study villages

A total of 643 participants were enrolled in 16 study villages (Table 1) (Fig. 1). The study population included 170 (75.6%) and 193 (46.2%) females in Enugu and Ogun state study villages, respectively. The median age was 51 (range: 18–80) in Enugu and 45 (range: 18–92) in Ogun state. All villages in Enugu state had received 26 rounds of ivermectin MDA. Seven of 10 villages surveyed in Ogun state had received between 11 and 14 rounds of ivermectin prior to our study, and two surveyed villages in Ogun were ivermectin-naïve at the time of our study. All surveys were conducted 8 to 12 months after the last round of ivermectin MDA.

**Table 1:** Demographic and intervention data of study villages

Variable	Enugu state	Ogun state
Total number of participants	225	418
Gender		
Male	55 (24.4%)	225 (53.8%)
Female	170 (75.6%)	193 (46.2%)
Gender ratio		
Male: Female	1:3	1.2:1
Median age (range)	51 (18–80)	45 (18–92)

### Onchocerciasis infection parameters

Table 2 shows nodule and skin Mf prevalence and geometric mean skin Mf counts in the study villages in Enugu and Ogun states. Nodule and skin Mf prevalence in Enugu villages surveyed ranged from 42 to 67%, and from 32 to 51%, respectively. However, skin Mf densities were quite low; geometric mean Mf/mg values ranged from 1.0 to 3.1. Onchocerciasis infection parameters were lower in surveyed villages in Ogun state where nodule and skin Mf prevalence ranged from 0 to 25% and from 0 to 28.6%, respectively. Geometric mean Mf/mg were less than 1 Mf/mg in all villages surveyed in Ogun (with the exception of one community, *Ibaro*, where the geometric mean Mf/mg value was 3.4).

**Table 2:** *O. volvulus* nodule, skin Mf prevalence, and density after several rounds of ivermectin mass drug administration

State	Study village	Baseline nodule prevalence^a,b^	Number of ivermectin MDA rounds	Number of participants	Nodule prevalence (95% CI)	Skin microfilaria prevalence (95% CI)	Geometric mean Mf/mg
Enugu							
	Amokwe	42.0	26	45	55.6 (44.2, 60.5)	51.1 (37.0, 65.0)	2.2
	Eziobodo	54.0	26	45	51.1 (37.0, 65.0)	33.3 (21.4, 48.0)	3.1
	Obinagu/Eziama	58.0	26	50	42.0 ( 29.4, 55.8)	32.0 (20.8, 45.9)	3.0
	Ugwuorie	42.0	26	30	46.7 (30.3, 63.8)	36.7 (21.9, 54.6)	1.0
	Umuezemanna	44.0	26	15	66.7, 41.5, 84.8)	46.7 (24.9, 69.8)	1.6
	Umunnakwe	44.0	26	40	62.5 (46.9, 75.6)	50.0 (35.2, 64.8)	2.2
Ogun							
	Abule Aje	4.8	14	41	0 (0.0, 10.5)	4.9 (0.6, 17.2)	0.3
	Abule Peter	4.8	14	43	0 (0.0, 10.0)	2.3 (0.0, 13.4)	0.2
	Adeaga	16.0	11	32	3.1 (0.0, 17.4	28.1 (15.5, 45.6)	0.4
	Ibara Afon	30.0	14	48	4.2 (0.4, 14.9)	6.3 (1.6, 17.6)	0.3
	Ibaro	12.0	14	42	9.5 (0.3, 22.8)	28.6 (17.1, 43.7)	3.4
	Idode	6.0	0	50	4.0 (0.4, 14.4)	20.0 (11.1, 33.1)	0.3
	Imomo	6.0	0	40	25.0 (13.1, 42.4)	15.0 (6.8, 29.6)	0.3
	Isara	22.0	3	32	10.7 (3.0, 28.2)	9.4 (2.6, 25.2)	0.3
	Olokemeji	16.0	11	28	10.9 (4.4, 23.6)	21.4 (9.9, 40.0)	0.4
	Olowo	16.0	11	46	3.7 (2.2, 6.1)	0.0 (0.0, 9.4)	0.0

^a^Baseline surveys (REMO) for Enugu state study villages were conducted in 1994

^b^Baseline surveys (REMO) for Ogun state study villages were conducted between 1995 and 2000

### Gender distribution of nodules and Mf in the study villages

Table 3 shows onchocerciasis infection parameters (nodule and skin Mf prevalence), stratified by gender. In Enugu state, there were no significant differences in nodule or skin Mf prevalence by gender (*p* = 0.056 and 0.161 for nodule and skin Mf, respectively). Also, there were statistically significant differences in nodule prevalence by gender in study villages in Ogun state (*p* = 0.021), but not for skin Mf (*p* = 0.817). Descriptive statistics of the number of individuals with nodules and skin Mf, and with either nodules or skin Mf are presented as Additional file 1.

**Table 3:** Gender distribution of nodules and skin microfilaria prevalence in the study villages

State	Gender	Number of participants	Nodule prevalence (95% CI)	Skin microfilaria prevalence (95% CI)
Enugu				
	Female	170	48.8 (41.4, 56.3)	37.7 (30.7, 45.1)
	Male	55	63.6 (50.4, 75.1)	50.9 (38.1, 63.6)
Ogun				
	Female	215	3.7 (1.8, 7.3)	14.0 (9.9, 19.3)
	Male	187	10.2 (0.7, 15.4)	12.8 (8.7, 18.5)

## Discussion

This study has provided new data on the current status of onchocerciasis in selected LGAs in endemic states in southeast and southwest Nigeria. Although all surveyed villages in Enugu state had received many rounds of ivermectin MDA (26 rounds over more than two decades), our results showed that nodule and skin Mf prevalences were still at meso-endemic levels. In contrast, onchocerciasis infection prevalences in villages surveyed in Ogun state were considerably lower than those in Enugu even though fewer rounds of ivermectin had been distributed in Ogun. This difference may be due to higher baseline infection prevalences and biting rates in Enugu.

Rapid epidemiological mapping of onchocerciasis (REMO) was conducted in 1994 in Enugu state. Some 27 years later, nodule prevalence remained largely unchanged in this setting. This may be because adult *O. volvulus* worms can live in subcutaneous nodules for about 15 years, and ivermectin does not kill adult worms [13, 14], [15]. The persistence of onchocerciasis in these study areas could be due to suboptimal compliance with MDA or to extremely high *Simulium* biting rates.

*O. volvulus* skin Mf densities are correlated with disease risk [2]. We do not have access to ivermectin MDA coverage and compliance data for villages in this study, and that is a limitation of our study. However, low skin Mf counts documented in this study are encouraging, because they suggest that MDA coverage and compliance have been high in the recent past in the surveyed villages which were considered by state health officials to be at high risk for persistence of onchocerciasis. They also suggest that ivermectin MDA is working well for disease control in these areas. On the other hand, these results also mean that the surveyed areas did not have enough heavily infected patients to populate our planned clinical trials of new treatments for onchocerciasis. That is good news for Ogun and Enugu states.

We found a statistically significant difference in nodule prevalence rates between males and females in our study population. This is consistent with results from other studies where males had higher nodule prevalences [16, 17]. These studies suggest that higher infection prevalences in males were due to their higher exposure to bites from the *Simulium* vector during outdoor activities such as farming and fishing [17, 18]. There were no significant differences in Mf prevalence by gender in our study. Differences that may have been present at baseline may have been obscured over time by ivermectin MDA.

The small number of adults sampled per village is a limitation of our study because this results in wide confidence intervals for estimates of infection prevalence by village. Also, the sampling population in this study differed from previous REMO surveys; REMO methodology restricted surveys to a random population of adult males whereas our study did not have any gender restrictions. This difference in sampling population makes it tricky to directly compare our results with results obtained at baseline surveys. Despite these limitations, our results clearly show that these study areas need to continue ivermectin MDA despite the long history of ivermectin distribution in the Enugu study villages and mostly low baseline nodule prevalences in the Ogun study villages. The 2016 WHO onchocerciasis elimination targets require the demonstration of very low infection prevalences in children [19]. Although we did not include children in our study, the infection rates recorded in adults in our study suggest that these study areas would not meet recommended MDA stopping criteria. Furthermore, mathematical modelling predictions suggest that for an evaluation unit to be considered to have interrupted transmission, skin Mf prevalence should be < 5.0% in 100% of the villages in an implementation unit or < 1.0% in at least 90% of villages [20].

In conclusion, this study has shown that onchocerciasis persists in the areas surveyed in two states in Nigeria despite many rounds of ivermectin MDA. Although *O. volvulus* transmission has been interrupted in some endemic foci in Africa with ivermectin MDA, this intervention is more effective for disease prevention than for elimination. Our results suggest that annual ivermectin alone may not be sufficient to eliminate transmission by 2030 in areas similar to those of our study villages in Enugu. Options to consider include more frequent MDA with ivermectin (with high treatment coverage and compliance), use of a more effective microfilaricide such as moxidectin [6, 21], distribution of an effective macrofilaricide (not yet available), or addition of vector control to complement mass drug administration.

## Supplementary Information

**Additional file 1: Table S1.** The presence of nodules and skin microfilaria (mf) at individual level in study population.

## Data Availability

All relevant data are contained within the manuscript.

## References

[1] Malecela MN, Ducker C (2021). A road map for neglected tropical diseases 2021–2030. Trans R Soc Trop Med Hyg.

[2] World Health Organization. Onchocerciasis: symptomatology, pathology, diagnosis. A. A. Buck, editor. Geneva: World Health Organization; 1974.

[3] Vlassoff C, Weiss M, Ovuga EB, Eneanya C, Nwel PT, Babalola SS (2000). Gender and the stigma of onchocercal skin disease in Africa. Soc Sci Med.

[4] Gemade EI, Jiya JY, Nwoke BE, Ogunba EO, Edeghere H, Akoh JI (1998). Human onchocerciasis: current assessment of the disease burden in Nigeria by rapid epidemiological mapping. Ann Trop Med Parasitol.

[5] World Health Organization. Conceptual and operational framework of onchocerciasis elimination with ivermectin treatment. Ouagadougou: African Programme for Onchocerciasis Control; 2010 2010. Contract No.: JAF16.6(II).

[6] Opoku NO, Bakajika DK, Kanza EM, Howard H, Mambandu GL, Nyathirombo A (2018). Single dose moxidectin versus ivermectin for Onchocerca volvulus infection in Ghana, Liberia, and the Democratic Republic of the Congo: a randomised, controlled, double-blind phase 3 trial. Lancet.

[7] Chavasse DC, Post RJ, Lemoh PA, Whitworth JA (1992). The effect of repeated doses of ivermectin on adult female Onchocerca volvulus in Sierra Leone. Trop Med Parasitol.

[8] Federal Ministry of Health. Neglected Tropical Diseases—Nigeria multi-year master plan 2015–2020 Abuja: Federal Ministry of Health; 2015.

[9] Okorie PN, Davies E, Ogunmola OO, Ojurongbe O, Saka Y, Okoeguale B (2015). Lymphatic filariasis baseline survey in two sentinel sites of Ogun state, Nigeria. Pan Afr Med J..

[10] Eneanya OA, Fronterre C, Anagbogu I, Okoronkwo C, Garske T, Cano J (2019). Mapping the baseline prevalence of lymphatic filariasis across Nigeria. Parasit Vectors.

[11] Fischer PU, King CL, Jacobson JA, Weil GJ (2017). Potential value of triple drug therapy with ivermectin, diethylcarbamazine, and albendazole (IDA) to accelerate elimination of lymphatic filariasis and onchocerciasis in Africa. PLoS Negl Trop Dis.

[12] Crosskey RW (1981). A review of Simulium damnosum s.l. and human onchocerciasis in Nigeria, with special reference to geographical distribution and the development of a Nigerian national control campaign. Tropenmed Parasitol..

[13] Awadzi K, Boakye DA, Edwards G, Opoku NO, Attah SK, Osei-Atweneboana MY (2004). An investigation of persistent microfilaridermias despite multiple treatments with ivermectin, in two onchocerciasis-endemic foci in Ghana. Ann Trop Med Parasitol.

[14] Dadzie Y, Amazigo UV, Boatin BA, Sékétéli A (2018). Is onchocerciasis elimination in Africa feasible by 2025: a perspective based on lessons learnt from the African control programmes. Infect Dis Poverty.

[15] Borsboom GJ, Boatin BA, Nagelkerke NJ, Agoua H, Akpoboua KL, Alley EW (2003). Impact of ivermectin on onchocerciasis transmission: assessing the empirical evidence that repeated ivermectin mass treatments may lead to elimination/eradication in West-Africa. Filaria J.

[16] Basáñez MG, Boussinesq M (1999). Population biology of human onchocerciasis. Philos Trans R Soc Lond B Biol Sci.

[17] Eyo J, Onyishi G, Ugokwe C (2013). Rapid epidemiological assessment of onchocerciasis in a tropical semi-urban community, Enugu State, Nigeria. Iran J Parasitol..

[18] Brabin L (1990). Factors affecting the differential susceptibility of males and females to onchocerciasis. Acta Leiden.

[19] World Health Organization (2016). Guidelines for stopping mass drug administration and verifying elimination of human onchocerciasis: criteria and procedures.

[20] Diawara L, Traoré MO, Badji A, Bissan Y, Doumbia K, Goita SF (2009). Feasibility of onchocerciasis elimination with ivermectin treatment in endemic foci in Africa: first evidence from studies in Mali and Senegal. PLoS Negl Trop Dis.

[21] Milton P, Hamley JID, Walker M, Basáñez MG (2020). Moxidectin: an oral treatment for human onchocerciasis. Expert Rev Anti Infect Therapy.

