# Supplementary material for: Persistence of onchocerciasis in villages in Enugu and Ogun states in Nigeria following many rounds of mass distribution of ivermectin
Source: BMC Infect Dis. 2022 Nov 10;22:832. doi: 10.1186/s12879-022-07811-7 (PMC9650792; doi:10.1186/s12879-022-07811-7)
Supplement: Supplementary file 1 — Additional file 1: Table S1. The presence of nodules and skin microfilaria (mf) at individual level in study population. [file 12879_2022_7811_MOESM1_ESM.docx]

Additional file 1: Association between the presence of nodule and skin microfilaria in participants in Enugu and Ogun state, Nigeria

The association between the presence of nodules and the presence of skin Mf at the individual level is presented in Supplementary 1. In Ogun State, less than 1% of participants presented with both nodule and skin Mf in the study villages. Whereas 12.2% of the total number of participants were positive for skin Mf but had no palpable nodules. Also, 5.98% total number of participants had nodules, but no skin mf was observed. In Enugu state, surveys were conducted between September and October 2020. Strict COVID-19 preventive measures were put in place to protect both field staff and survey participants. Therefore, to reduce person-to-person contact, skin Mf snipping was restricted only to participants with palpable nodules. Our results show that 40.89% of study participants were positive for both nodules and skin Mf, whereas 44.89% did not have palpable nodules, but were positive for Skin Mf.

Table S1: The Presence of Nodules and Skin Microfilaria (mf) at Individual level in Study Population

| **State** | **Study Village** | **Number of participants examined** | **Number of individuals with nodule and skin Mf** | **Number of individuals with nodule but no skin Mf** | **Number of individuals without nodule but skin Mf** | **Number of individuals without nodule and skin Mf** | **P value** |
| --- | --- | --- | --- | --- | --- | --- | --- |
| Enugu | Amaokwu | 45 | 23 (51.11) | 8(17.78) | NA | 14 (31.11) | - |
|  | Eziobodo | 45 | 15 (33.33) | 8 (17.78) | NA | 22 (48.89) | - |
|  | Obinagu/Eziama | 50 | 16 (32.0) | 5 (10.0) | NA | 29 (58.0) | - |
|  | Ugwuorie | 30 | 11 (36.67) | 3 (10.0) | NA | 16 (53.33) | - |
|  | Umuezemanna | 15 | 7 (46.67) | 3 (20.0) | NA | 5 (33.33) | - |
|  | Umunnakwe | 40 | 20 (50.0) | 5 (12.5) | NA | 15 (37.5) | - |
|  | **Total** | **225** | **92 (40.89)** | **32 (14.22)** | **NA** | **101 (44.89)** | **-** |
|  |  |  |  |  |  |  |  |
| Ogun | Abule Aje | 41 | 0 (0.0) | 0(0.0) | 2 (4.87) | 39 (95.12) | - |
|  | Abule Peter | 43 | 0 (0.0) | 0 (0.0) | 1 (2.32) | 42 (97.7) | - |
|  | Adeaga | 32 | 0 (0.0) | 1 (3.13) | 9 (28.13) | 22 (68.75) | 0.525 |
|  | Ibara Afon | 48 | 0 (0.0) | 2 (4.16) | 3 (6.25) | 43 (89.59) | 0.709 |
|  | Ibaro | 42 | 1 (2.38) | 3 (7.14) | 11(26.19) | 27 (64.29) | 0.892 |
|  | Idode | 50 | 0 (0.0) | 2 (4.0) | 10 (20.0) | 38 (76.0) | 0.471 |
|  | Imomo | 40 | 0 (0.0) | 3 (7.5) | 6 (15.0) | 31 (77.7) | 0.449 |
|  | Isara | 32 | 1(3.13) | 6(18.75) | 2 (6.25) | 23 (71.88) | 0.614 |
|  | Olokemeji | 28 | 1(3.57) | 2 (7.14) | 5 (17.86) | 20 (71.42) | 0.595 |
|  | Olowo | 46 | 0 (0.0) | 5(10.87) | 0 (0.0) | 41(89.13) | - |
|  | **Total** | **401** | **3 (0.75)** | **24 (5.98)** | **49 (12.21)** | **325 (81.05)** | **0.766** |

**NA=Not Applicable. Only participants with nodules were screened for skin Mf.**
